# Association between the uric acid to high-density lipoprotein cholesterol ratio and diabetic kidney disease in U.S. adults with type 2 diabetes: A cross-sectional study of NHANES 2009 to 2018

**DOI:** 10.1097/MD.0000000000051033

**Published:** 2026-10-16

**Authors:** Ruolan Tan, Wei Tan, Yulin Hu

**Affiliations:** a Departments of Nephrology, The Affiliated Yongchuan Hospital of Chongqing Medical University, Chongqing, China; b College of Medical Laboratory, Dalian Medical University, Dalian, Liaoning, China.

**Keywords:** diabetic kidney disease, HDL cholesterol, NHANES, UHR, United States, uric acid

## Abstract

The uric-acid-to- high-density lipoprotein-cholesterol ratio (UHR) combines 2 circulating biomarkers related to metabolic and inflammatory processes. Its association with prevalent diabetic kidney disease (DKD) was examined in U.S. adults with type 2 diabetes mellitus. This study aimed to examine the cross-sectional association between UHR and prevalent DKD and assess its incremental discrimination beyond the specified covariate model. We analyzed 4010 adults with type 2 diabetes mellitus from NHANES 2009 to 2018 using the complex survey design. DKD was defined as urinary albumin-to-creatinine ratio ≥30 mg/g and/or estimated glomerular filtration rate <60 mL/min/1.73 m^2^, calculated using the 2009 chronic kidney disease epidemiology collaboration equation without its race coefficient. Strict DKD required both abnormalities; severe DKD required urinary albumin-to-creatinine ratio ≥300 mg/g. Model 3 was the primary confounder-adjusted model, and model 4 added laboratory indicators as an extended sensitivity analysis. Supplementary analyses assessed nonlinearity, discrimination and graphical calibration. Exploratory unweighted category-based net reclassification improvement used 500 bootstrap replicates and percentile confidence intervals. Of 4010 participants, 1576 had study-defined DKD; the weighted prevalence was 35.4%. In model 3, the highest versus lowest ln-UHR quartile had an odds ratio of 2.43 (95% confidence interval 1.75–3.38) for original DKD, 4.55 (2.81–7.35) for strict DKD and 2.82 (1.57–5.06) for severe DKD. Estimates were attenuated in model 4, with the severe-DKD contrast no longer meeting the 0.05 threshold. The laboratory-adjusted spline indicated nonlinearity (*P* < .001). In the separate laboratory-inclusive discrimination analysis, adding ln-UHR increased apparent area under the curve from 0.75 to 0.76; the unweighted net reclassification improvement was 1.9% (95% confidence interval 0.8%–3.1%). Higher UHR was associated with prevalent DKD after adjustment for the selected potential confounders. Supplementary analyses showed a small increase in apparent discrimination. These cross-sectional findings require prospective investigation and independent external validation before use for future risk prediction or clinical screening.

## 1. Introduction

Chronically high blood glucose levels are a hallmark of diabetes, a metabolic illness, with a complex pathogenesis involving both insulin synthesis and action. As the social economy develops rapidly, the number of diabetics has dramatically increased. This phenomenon is closely related to the improvement of living standards and the continuous rise in obesity rates. It is projected that by 2035, 592 million people worldwide are expected to have diabetes.^[1]^ The clinical symptoms of diabetes are diverse, mainly including hyperglycemia, increased thirst (polydipsia), increased urination (polyuria), and increased hunger (polyphagia). Based on variations in etiology and pathophysiology, diabetes can be classified as either type 1 diabetes or type 2 diabetes (T2DM). The most prevalent metabolic disease in the world, T2DM, is directly linked to insulin resistance and the increasing deterioration of pancreatic β-cell function. Its development is typically linked to obesity, physical inactivity, genetic factors, and unhealthy lifestyle habits.^[2]^ T2DM is a chronic, noninfectious multisystem disease characterized by chronic hyperglycemia, which affects microvessels and can contribute to diabetic kidney disease (DKD), diabetic retinopathy, and neuropathy.^[3,4]^ As a principal microvascular sequela of diabetes mellitus, DKD exerts profound effects on morbidity trajectories through persistent albuminuria (≥30 mg/g creatinine) and/or gradual decline in glomerular filtration rate < 60 mL/min/1.73m^2^, ultimately diminishing health-related quality of life indices.^[5]^ DKD has a subtle onset and is difficult to detect, yet it has a profound impact on renal function and this condition represents a predominant etiological factor in the progression to chronic kidney disease (CKD) and subsequent end-stage renal disease.^[6,7]^ The increasing mortality and medical expenses associated with this disease have exerted tremendous pressure on the global healthcare system. Therefore, preventing the development of end-stage renal disease and prolonging the life expectancy of patients depend on the early detection of DKD.

With the in-depth research into the pathogenesis of DKD, the pathophysiological role of uric acid (UA) has increasingly attracted the attention of the academic community. Upon completion of purine metabolism, UA exhibits significant correlations with DKD pathogenesis through abnormal metabolic pathways.^[8,9]^ Homeostatic disruption of UA metabolism, manifesting as hyperuricemia, emerges as a pathogenic precursor to multisystem dysregulation. This crystalloid imbalance demonstrates significant clinical ramifications across cardiometabolic pathologies, notably potentiating diabetic progression, adiposity-related disorders, hypertensive sequelae, and systemic metabolic dysfunction syndromes.^[10]^ UA exerts nephrotoxic effects through multiple pathways, with chronic hyperuricemia directly contributing to renal functional decline.^[11]^ Therefore, high UA levels in patients with T2DM can affect the course of renal disease and are a high-risk factor for the onset of early kidney disease. Uric acid exhibits antioxidant properties at physiological concentrations, but at elevated levels, experimental evidence indicates that UA triggers oxidative stress and inflammatory responses, pivotal pathways in diabetic kidney disease pathogenesis. Elevated serum UA concentrations have been consistently linked to greater odds of prevalent DKD in cross-sectional studies.^[9,12,13]^

The liver and small intestine are the primary sites for the synthesis of high-density lipoprotein cholesterol (HDL-C). It is involved in promoting cholesterol reverse transport, assisting in the removal of cholesterol from peripheral tissues and preserving the equilibrium of the body’s lipid metabolism. Variations in HDL-C levels are one of the important biomarkers for assessing the risk of cardiovascular and cerebrovascular diseases.^[14]^ HDL-C exerts its anti-atherosclerotic protective effects by delivering cholesterol to the liver for metabolism from peripheral tissues. Therefore, it is often referred to as “good cholesterol.”^[15]^ Evidence indicates that HDL-C levels are intimately linked to DKD development and occurrence. HDL-C is not only involved in lipid metabolism but additionally possesses anti-inflammatory, antioxidant, and protective effects on vascular endothelial function. Under diabetic conditions, abnormal lipid metabolism is one of the causes of DKD, and variations in HDL-C levels may reflect this pathological process. Emerging evidence elucidates intricate associations between HDL-C dyslipidemia profiles and the pathogenesis of DKD, particularly through modulating inflammatory cascades and endothelial dysfunction mechanisms.^[16,17]^

A new biomarker for evaluating inflammation and metabolic function is the uric acid-to-HDL-cholesterol ratio (UHR), which measures both UA and blood lipid levels. Research has found that compared to healthy individuals, significantly elevated UHR levels are found in individuals with nonalcoholic fatty liver disease (NAFLD), and these levels are positively connected with metabolic and inflammatory indicators such body weight, body mass index (BMI), triglycerides, transaminase, and fasting blood glucose.^[18]^ Additionally, a cross-sectional investigation showed that serum UHR might predict death from cardiovascular disease (CVD) and all causes.^[19]^ In a study focusing on individuals suffering from chronic complete occlusion, have identified elevated the UHR as an independent predictor of major adverse cardiovascular events in diabetic populations.^[20]^ Additionally, UHR elevation demonstrates significant associations with diabetic microvascular complications, including nephropathy progression, ischemic cardiac pathology, and impaired insulin sensitivity.^[21–23]^ This study examines the association between UHR and prevalent DKD in the American population; it does not evaluate early detection, prevention or disease management outcomes. Although previous studies based on Chinese populations have suggested an association between UHR and DKD, their generalizability remains uncertain due to limitations such as single-center designs, insufficient sample representativeness, and lack of ethnic diversity. Using the cross-sectional National Health and Nutrition Examination Survey (NHANES) data, we examined the UHR–DKD association in a multi-ethnic U.S. sample and evaluated discrimination for prevalent DKD. This analysis is not a prospective or external validation study.

## 2. Methods

### 2.1. Analyze the individuals

The National Center for Health Statistics (NCHS) performed the NHANES, which provided the data used in this investigation. NHANES is a thorough survey created to assess possible health risk factors in the US population that is not hospitalized. It includes a broad variety of variables, including demographics, socioeconomic status, educational attainment, dietary habits, lifestyle behaviors such as smoking and alcohol consumption, exposure to environmental pollutants, and preexisting medical conditions. In order to create a nationally representative cohort and guarantee population-level generalizability, NHANES used a multistage probability sampling strategy with stratification. The Centers for Disease Control and Prevention’s Institutional Review Board formally approved the protocol, and all individuals provided written consent before being enrolled. Full methodological specifications are accessible via the official NHANES portal. The NHANES protocols were approved by the NCHS Research Ethics Review Board. All participants gave written informed consent. As the data are de-identified and publicly available, additional institutional review board approval for this secondary analysis was not required.This study pooled 5 NHANES cycles (2009–2018) and accounted for the complex sampling design provided by NCHS. Primary sampling units (SDMVPSU), strata (SDMVSTRA), and 5-cycle adjusted weights (WTMEC2YR/5) were incorporated into all descriptive, regression, subgroup, and receiver operating characteristic (ROC) analyses to ensure national representativeness.

### 2.2. Data and sample sources

Initially, 49,693 people participated in our study spanning 5 cycles, from 2009 to 2018. However, we excluded participants who were younger than 20 years old (n = 20,858), pregnant (n = 315), had missing information on serum UA and high-density lipoprotein to calculate UHR (n = 2852), had missing information on urinary albumin/creatinine ratio (UACR) and estimated glomerular filtration rate (eGFR) (n = 364), had cancer (n = 2356), and did not have T2DM (n = 18,938) based on the study’s exclusion criteria. Figure 1 shows the specifics of the final analysis, which comprised 4010 people who fulfilled the predetermined criteria. Of these, 2434 participants did not have DKD, whereas 1576 participants did. Missingness was highest for poverty income ratio (PIR) (n = 431), BMI (n = 65), globulin (n = 15), glycated hemoglobin (HbA1c) (n = 6), and aspartate aminotransferase (n = 1). To evaluate missing-data impact, we performed multiple imputation by chained equations (m = 5 imputations and 20 iterations). The imputation model included all analysis variables (exposure, outcome, and covariates) plus design variables (SDMVSTRA and SDMVPSU) to preserve survey structure; sampling weights were not included in the imputation equation following recommended practice. Each completed dataset was analyzed with full survey weighting, and estimates were pooled using Rubin rules. Pooled estimates differed by <5% from complete-case results, indicating robustness.

**Figure 1.**
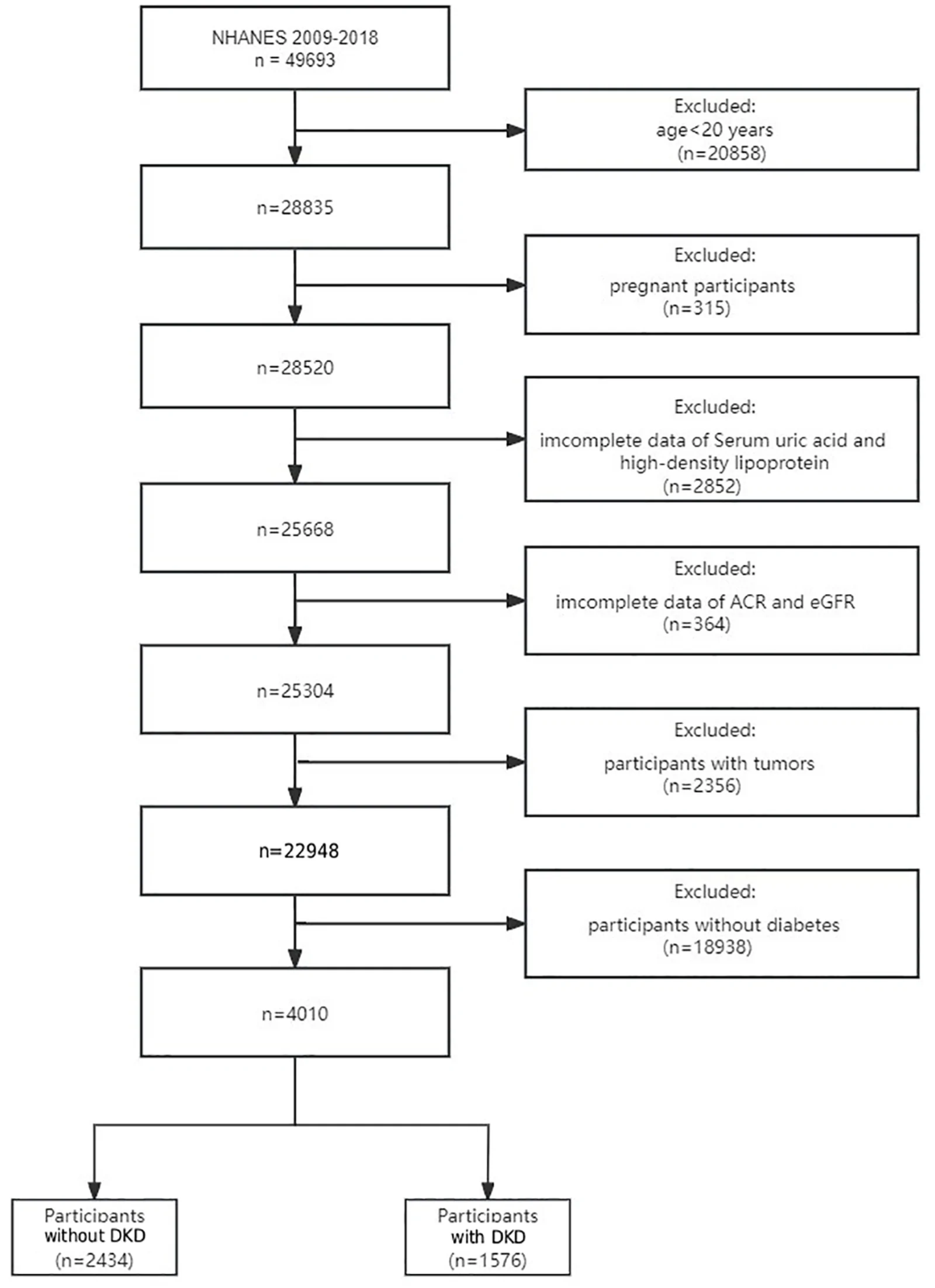
Flowchart of participant selection from NHANES 2009 to 2018. ACR = albumin-to-creatinine ratio, DKD = diabetic kidney disease, eGFR = estimated glomerular filtration rate, NHANES = National Health and Nutrition Examination Survey.

### 2.3. Definition of UHR and DKD

In the early morning fasting state, researchers collected blood samples from participants to determine the levels of HDL and UA. Subsequently, UA and HDL were divided, and the result was multiplied by 100 to determine the UHR. UHR is defined as the ratio of uric acid to high-density lipoprotein cholesterol. $UHR=\frac{Serum\;UA\;\left(mg/dL\right)}{HDL\text{\textasciitilde{}}\text{-}C\left(mg/dL\right)}\times 100\%$

Diabetes was identified based on a prior diagnosis by medical professionals, fasting plasma glucose ≥7.0 mmol/L, HbA1c ≥6.5%, use of diabetes medications, or insulin therapy.^[24,25]^According to the Kidney Disease: Improving Global Outcomes 2021 guidelines, DKD was defined by the presence of diabetes mellitus with a UACR ≥ 30 mg/g and/or an eGFR < 60 mL/min/1.73m^2^.^[25–27]^ Albuminuria status was determined through UACR quantification. Renal filtration capacity was assessed via eGFR calculations employing the chronic kidney disease epidemiology collaboration (CKD-EPI) equation. The gender-specific computation followed established parameters: In female, eGFR = 144 × (sCr/0.7)^−0.329^ × 0.993^Age^ if sCr ≤ 0.7 mg/dL and 144 × (sCr/0.7)^−1.209^ × 0.993^Age^ if sCr is >0.7 mg/dL. In male, eGFR = 141 × (sCr/0.9) ^−0.411^ × 0.993^Age^ if sCr is ≤ 0.9 mg/dL and 141 × (sCr/0.9) ^−1.209^ × 0.993^Age^ if sCr is > 0.9 mg/dL.^[28]^ The eGFR was calculated from serum creatinine using the 2009 CKD-EPI equation with sex-specific coefficients, consistent with NHANES 2009 to 2018 data documentation. The original race multiplier of 1.159 was not applied to any participant. eGFR was calculated rather than taken from a precomputed NHANES eGFR variable; this implementation was not the 2021 CKD-EPI equation. The race multiplier was omitted because race-based coefficient adjustment has been discouraged in current eGFR estimation practice and its omission provides a uniform, race-free implementation across all participants.Two sensitivity definitions were added: DKD strict (UACR ≥ 30 and eGFR < 60) and DKD severe: macro-albuminuria (UACR ≥ 300 mg/g).

### 2.4. Definition of other variables

A directed acyclic graph (DAG) was constructed in DAGitty to summarize hypothesized relationships between UHR, DKD, and the measured covariates (Fig. S1, Supplemental Digital Content 1). The DAG guided consideration of potential confounding and downstream processes; these causal roles are assumptions rather than findings established by the cross-sectional data.

The sequential adjustment framework included demographic and socioeconomic characteristics (age, sex, race/ethnicity, education, and PIR), behavioral and metabolic characteristics (smoking, alcohol use, BMI, and HbA1c), hypertension, and additional laboratory measurements (alanine transaminase [ALT], albumin, blood urea nitrogen [BUN], and globulin). The previously reported variance inflation factors were below 5. Covariate definitions were based on NHANES questionnaire, examination, and laboratory information.

BUN, albumin, and globulin reflect kidney function, inflammation, or nutritional status, while ALT provides information on related hepatic and metabolic characteristics. These measurements were therefore considered separately from the primary adjustment set because their roles can include confounding, downstream processes, or consequences of existing disease.

### 2.5. Statistical analysis

Analyses were conducted in R version 4.4 (R Core Team, R Foundation for Statistical Computing) and EmpowerStats version 2.0 (X&Y Solutions, Inc.). Tests were 2-sided, with *P* < .05 used as the statistical significance threshold. A Taylor-linearized survey design object was specified with the R survey package using SDMVPSU, SDMVSTRA, and WTMEC2YR/5. Weighted means and standard errors were estimated with svymean(), and categorical proportions and logit-transformed confidence intervals with svyciprop(). Group comparisons used Rao–Scott-adjusted chi-square tests for categorical variables and design-based Kruskal–Wallis tests for continuous variables. Continuous characteristics are reported as weighted means (standard errors) and categorical characteristics as weighted counts (percentages).

Participants were divided into ln-UHR quartiles (*Q*1–*Q*4). Survey-weighted logistic regression models were fitted sequentially: model 1 included ln-UHR alone; model 2 adjusted for age, sex, race/ethnicity, education, PIR, smoking, alcohol use, BMI, and HbA1c; model 3 additionally included hypertension; and model 4 additionally included albumin (ALB), ALT, BUN, and globulin (GLB). Model 3 was selected as the primary confounder-adjusted association model for all 3 DKD definitions. Guided by the hypothesized relationships in the DAG (Fig. S1, Supplemental Digital Content 1), ALB, ALT, BUN, and GLB were considered to have uncertain causal roles, including potential confounding and processes downstream of UHR or DKD. Model 4 was therefore presented as an extended sensitivity analysis assessing the primary association after additional laboratory adjustment, rather than as the primary model or a formal mediation analysis.

Supplementary analyses examined nonlinearity using a survey-weighted restricted cubic spline with the model 4 adjustment set and 4 knots at the 5th, 35th, 65th, and 95th percentiles. The continuous model 4 coefficient summarizes a linear-logit contrast and does not imply a constant association across the spline. Exploratory subgroup analyses considered sex, age (<60 or ≥ 60 years), education, BMI (<25, 25–<30 or ≥30 kg/m^2^), PIR (<1.3, 1.3–<3.5 or ≥3.5), hypertension, lifetime smoking of at least 100 cigarettes, and HbA1c (<7% or ≥7%). The subgroup-specific adjustment set is listed in Table 3; these estimates are supplementary and are not relabeled as model 3 estimates. Interaction tests were not adjusted for multiple comparisons.

**Table 3:** Subgroup analysis of the relationship between ln-UHR and DKD in participants with type 2 diabetes mellitus.

Subgroup	OR (95% CI)	*P*	*P* for interaction
Sex			.266
Male	2.11 (1.41–3.16)	**<.001**	
Female	1.61 (0.94–2.75)	.089	
Age (yr)			.789
<60	1.52 (0.90–2.55)	.120	
≥60	2.12 (1.31–3.42)	**.003**	
Education level			.545
Less than high school	1.95 (1.34–2.85)	**<.001**	
High school or GED	1.97 (1.13–3.45)	**.020**	
Above high school	1.78 (1.03–3.05)	**.042**	
BMI level			.456
<25	2.55 (1.30–5.01)	**.009**	
≥25–<30	1.27 (0.70–2.34)	.435	
≥30	2.01 (1.26–3.23)	**.005**	
PIR level			.894
<1.3	1.79 (1.12–2.85)	**.017**	
≥1.3–<3.5	1.76 (1.17–2.66)	**.009**	
≥3.5	2.04 (0.73–5.74)	.181	
Smoked at least 100 cigarettes in life			.186
No	2.28 (1.36–3.82)	**.003**	
Yes	1.50 (1.05–2.14)	**.031**	
Hypertension			.081
No	2.96 (1.73–5.06)	**<.001**	
Yes	1.57 (1.04–2.38)	**.036**	
HbA1c			.805
<7	1.76 (1.06–2.92)	**.034**	
≥7	1.91 (1.34–2.73)	**<.001**	

Age, sex, race, education, hypertension, BMI, PIR, ALB, ALT, GLB, and BUN were adjusted. In the subgroup analyses, the models were not adjusted for the stratification variable itself. ORs compare the highest (*Q*4) versus lowest (*Q*1) ln-UHR quartile within each subgroup.

The bold values indicate statistically significant results (*P* < .05).

ALB = albumin, ALT = alanine aminotransferase, BMI = body mass index, BUN = blood urea nitrogen, CI = confidence interval, DKD = diabetic kidney disease, GED = General Educational Development, GLB = globulin, HbA1c = glycated hemoglobin, ln UHR = the uric acid to high-density lipoprotein cholesterol ratio index was logarithmically transformed using the natural logarithm(ln), OR = odds ratio, PIR = poverty income ratio.

Discrimination and calibration were evaluated as supplementary analyses of prevalent DKD. For this separate model comparison, the base model included the model 4 covariates without ln-UHR, and the extended model added ln-UHR; the change in the primary association model did not change this laboratory-inclusive comparison. Regression fitting, ROC analysis, and graphical calibration retained the NHANES sampling weights, strata, and primary sampling units. ROC analysis included 500-replicate bootstrap internal validation. Calibration was assessed primarily from the visual agreement between mean estimated probabilities and observed DKD proportions in survey-weighted probability deciles, together with the apparent and bootstrap bias-corrected curves. The Hosmer–Lemeshow test was reported as a secondary check (supplementary goodness-of-fit assessment), not as the principal evidence of calibration.

An exploratory, unweighted category-based net reclassification improvement (NRI) compared the base and extended models. The categories were <10%, 10%–20%, and >20% estimated probability of prevalent DKD. Upward and downward reclassification denoted movement to a higher or lower category after adding ln-UHR; changes within a category contributed zero. Using the conventional participant-count formulation, NRI was calculated as the net proportion moving upward among participants with DKD plus the net proportion moving downward among participants without DKD:


$$\begin{array}{c} NRI=(nup,D-ndown,D)/nD+(ndown,N-nup,N)/nN. \end{array}$$

Here, n_D and n_N are the unweighted numbers of participants with and without DKD in the common model-comparison sample; the numerators count category movements within each group. NHANES sampling weights were not applied to these component proportions. The estimate and confidence limits were multiplied by 100 and reported as percentages.

The 95% confidence interval (CI) was obtained using unweighted bootstrap resampling (500 replicates, percentile method). The NRI was calculated for each replicate using the same probability categories, and the 2.5th and 97.5th percentiles of the bootstrap NRI distribution provided the lower and upper limits. The unweighted formulation was used for exploratory reclassification within the analyzed sample, rather than design-based estimation of population-level NRI. Its interval does not incorporate NHANES stratification or clustering; the regression, ROC, and graphical calibration analyses retained the survey design. The probability categories do not represent a future prediction horizon or validated clinical screening thresholds.

## 3. Results

### 3.1. Characteristics of participants

The eligible sample comprised 4010 participants, including 1576 with and 2434 without study-defined DKD. Table 1 presents the survey-weighted participant characteristics. The weighted population totals were 8,655,815 for participants with DKD and 15,803,235 for those without DKD, representing 24,459,050 U.S. adults with type 2 diabetes. These Table 1 totals correspond to a weighted DKD prevalence of 35.4%.

**Table 1:** Weighted baseline characteristics of U.S. adults with type 2 diabetes by diabetic kidney disease status, NHANES 2009 to 2018.

Characteristics (N)	Non-DKD (N = 15,803,235*)	95% CI	DKD (N = 8655,815*)	95% CI	*P* value†
Gender					.6
Male	8,443,606 (53%)	50%–57%	4,506,329 (52%)	48%–56%	
Female	7,359,629 (47%)	43%–50%	4,149,486 (48%)	44%–52%	
Race					.058
Mexican American	1,882,415 (12%)	9.3%–15%	936,449 (11%)	8.3%–14%	
Other Hispanic	1,138,482 (7.2%)	5.8%–8.8%	528,481 (6.1%)	4.6%–8.0%	
Non-Hispanic White	8,873,087 (56%)	52%–60%	4,744,568 (55%)	50%–59%	
Non-Hispanic Black	2,207,238 (14%)	12%–17%	1,509,536 (17%)	15%–21%	
Other races	1,702,012 (11%)	9.0%–13%	936,781 (11%)	8.8%–13%	
Education level					.018
Less than high school	3,400,531 (22%)	19%–24%	2,265,825 (26%)	24%–29%	
High school or GED	3,785,097 (24%)	21%–27%	2,296,505 (27%)	23%–30%	
Above high school	8,617,607 (55%)	51%–58%	4,093,485 (47%)	43%–51%	
PIR level					<.001
<1.3	3,662,064 (23%)	21%–25%	2,568,703 (30%)	27%–33%	
≥1.3–<3.5	6,537,777 (41%)	39%–44%	3,898,688 (45%)	42%–48%	
≥3.5	5,603,394 (35%)	32%–39%	2,188,425 (25%)	21%–30%	
BMI level					.042
<25	1,617,477 (10%)	8.7%–12%	913,000 (11%)	8.8%–13%	
≥25–<30	4,138,046 (26%)	23%–29%	1,890,383 (22%)	19%–24%	
≥30	10,047,711 (64%)	60%–67%	5,852,432 (68%)	64%–71%	
Age	5,905,464 (37%)	34%–41%	5,493,367 (63%)	59%–67%	<.001
ALB	4.18 (0.01)	4.2–4.2	4.06 (0.01)	4.0–4.1	<.001
ALT	29 (1)	28–30	27 (1)	24–30	<.001
BUN	14 (0)	14–14	19 (0)	19–20	<.001
GLB	2.91 (0.02)	2.9–2.9	3.07 (0.02)	3.0–3.1	<.001
HbA1c	6,534,573 (41%)	38%–44%	4,547,446 (53%)	49%–56%	<.001
Drinking	1.91 (0.05)	1.8–2.0	1.71 (0.07)	1.6–1.8	<.001
Hypertension	9,111,610 (58%)	55%–60%	6,311,029 (73%)	70%–76%	<.001
Smoked at least 100 cigarettes in life	7,656,214 (48%)	45%–52%	4,524,822 (52%)	49%–56%	.2

Weighted means (SE) and weighted counts (%) are presented; 95% confidence intervals were computed using survey-design corrected standard errors (Rao–Scott adjustment for categorical variables). Complex sampling design accounted for via primary sampling units (SDMVPSU), strata (SDMVSTRA), and 5-cycle adjusted weights (WTMEC2YR/5). *P* values were obtained using Rao–Scott-adjusted chi-square tests for categorical variables and design-based Kruskal–Wallis tests for continuous variables. Missing data excluded. Weighted totals: Non-DKD ≈ 15.8 million, DKD ≈ 8.7 million (raw N = 4010). Categories sum to weighted population under NHANES design. Design effects ranged 1.15–1.68 (median 1.32).

ALB = albumin, ALT = alanine aminotransferase, BMI = body mass index, BUN = blood urea nitrogen, CI = confidence interval, DKD = diabetic kidney disease, GED = General Educational Development, GLB = globulin, HbA1c = glycated hemoglobin, NHANES = National Health and Nutrition Examination Survey, PIR = poverty income ratio.

* n (%); mean (SE).

† Pearson *X*^2^: Rao and Scott adjustment; Design-based Kruskal–Wallis test.

Compared with participants without DKD, participants with DKD had a larger proportion with less than high-school education (26% vs 22%; overall education comparison *P* = .018) and a PIR below 1.3 (30% vs 23%; overall PIR comparison *P* < .001). The BMI-category distribution differed between groups (*P* = .042, with 68% of participants with DKD and 64% of those without DKD having a BMI ≥30 kg/m^2^. The overall comparisons for sex (*P* = .6, race/ethnicity (*P* = .058, and lifetime smoking of at least 100 cigarettes (*P* = .2 were not statistically significant.

Hypertension was more frequent in participants with DKD than in those without DKD (73% vs 58%; *P* < .001). Weighted mean albumin (4.06 vs 4.18) and ALT (27 vs 29) were lower in the DKD group, whereas BUN (19 vs 14) and globulin (3.07 vs 2.91) were higher (all *P* < .001).

### 3.2. The association between UHR and DKD

Table 2 presents the sequentially adjusted associations. In the primary analysis (model 3), the *Q*4-versus-*Q*1 odds ratio (OR) for original DKD was 2.43 (95% CI 1.75–3.38; *P* < .001). The corresponding estimates under the alternative definitions were 4.55 (95% CI 2.81–7.35; *P* < .001) for strict DKD and 2.82 (95% CI 1.57–5.06; *P* = .001) for severe DKD.

**Table 2:** Association between ln-UHR and DKD in patients with type 2 diabetes mellitus.

Variables	Model 1		Model 2		Model 3		Model 4	
	OR (95% CI)	*P*	OR (95% CI)	*P*	OR (95% CI)	*P*	OR (95% CI)	*P*
DKD								
Ln-UHR classification								
Quartile 1	1.00 (reference)		1.00 (reference)		1.00 (reference)		1.00 (reference)	
Quartile 2	1.25 (0.98–1.59)	.081	1.21 (0.91–1.60)	.186	1.22 (0.92–1.61)	.172	1.12 (0.83–1.50)	.458
Quartile 3	1.42 (1.10–1.83)	**.009**	1.58 (1.16–2.14)	**.005**	1.56 (1.15–2.12)	**.006**	1.41 (1.05–1.90)	**.026**
Quartile 4	2.27 (1.81–2.86)	**<.001**	2.49 (1.79–3.44)	**<.001**	2.43 (1.75–3.38)	**<.001**	1.84 (1.28–2.64)	**.002**
DKD strict								
Ln-UHR classification								
Quartile 1	1.00 (reference)		1.00 (reference)		1.00 (reference)		1.00 (reference)	
Quartile 2	1.56 (1.00–2.42)	.053	1.56 (0.97–2.53)	.074	1.57 (0.97–2.54)	.070	1.39 (0.83–2.31)	.216
Quartile 3	1.92 (1.25–2.94)	**.004**	2.41 (1.53–3.77)	**<.001**	2.37 (1.50–3.74)	**<.001**	2.05 (1.26–3.35)	**.005**
Quartile 4	3.55 (2.34–5.38)	**<.001**	4.71 (2.90–7.66)	**<.001**	4.55 (2.81–7.35)	**<.001**	2.78 (1.61–4.81)	**<.001**
DKD severe								
Ln-UHR classification								
Quartile 1	1.00 (reference)		1.00 (reference)		1.00 (reference)		1.00 (reference)	
Quartile 2	1.28 (0.72–2.28)	.400	1.21 (0.66–2.21)	.539	1.20 (0.66–2.20)	.549	1.07 (0.57–2.04)	.831
Quartile 3	1.20 (0.70–2.06)	.499	1.24 (0.72–2.15)	.440	1.21 (0.70–2.10)	.503	1.02 (0.57–1.84)	.941
Quartile 4	3.11 (1.79–5.40)	**<.001**	2.97 (1.66–5.31)	**<.001**	2.82 (1.57–5.06)	**.001**	1.93 (1.00–3.71)	.054

Model 1: Crude.

Model 2: Adjust: gender, race, education, smoke, age, PIR, BMI, HbA1c, and drinking.

Model 3:Adjust: gender, race, education, smoke, age, PIR, BMI, HbA1c, drinking, and hypertension.

Model 4: Adjust: gender, race, education, smoke, age, PIR, BMI, HbA1c, drinking, hypertension, ALB, ALT, BUN, and GLB.

Model 3 was the primary confounder-adjusted model. Model 4 was an extended sensitivity analysis adjusting for potential mediators (ALB, ALT, BUN, and GLB).

The bold values indicate statistically significant results (*P* < .05).

ALB = albumin, ALT = alanine aminotransferase, BMI = body mass index, BUN = blood urea nitrogen, CI = confidence interval, DKD = diabetic kidney disease, GLB = globulin, HbA1c = glycated hemoglobin, ln UHR = the uric acid to high-density lipoprotein cholesterol ratio index was logarithmically transformed using the natural logarithm(ln), OR = odds ratio, PIR = poverty income ratio.

In the extended sensitivity analysis (model 4), the *Q*4-versus-*Q*1 ORs were 1.84 (95% CI 1.28–2.64; *P* = .002) for original DKD, 2.78 (95% CI 1.61–4.81; *P* < .001) for strict DKD, and 1.93 (95% CI 1.00–3.71; *P* = .054) for severe DKD. Thus, all 3 estimates were attenuated after laboratory adjustment, and the severe-DKD contrast did not meet the 2-sided 0.05 threshold. The attenuation is compatible with the hypothesized downstream placement of these indicators in a UHR–DKD pathway, but the model comparison does not establish mediation or quantify a mediated effect.

In the supplementary continuous model 4 analysis, each 1-standard deviation increase in ln-UHR had a reported OR of 1.42 (95% CI 1.27–1.59). The model 4 restricted cubic spline showed nonlinearity (*P* for overall association <.001; *P* for nonlinearity <.001; Fig. 2). These are laboratory-adjusted supplementary results, not model 3 estimates.

**Figure 2.**
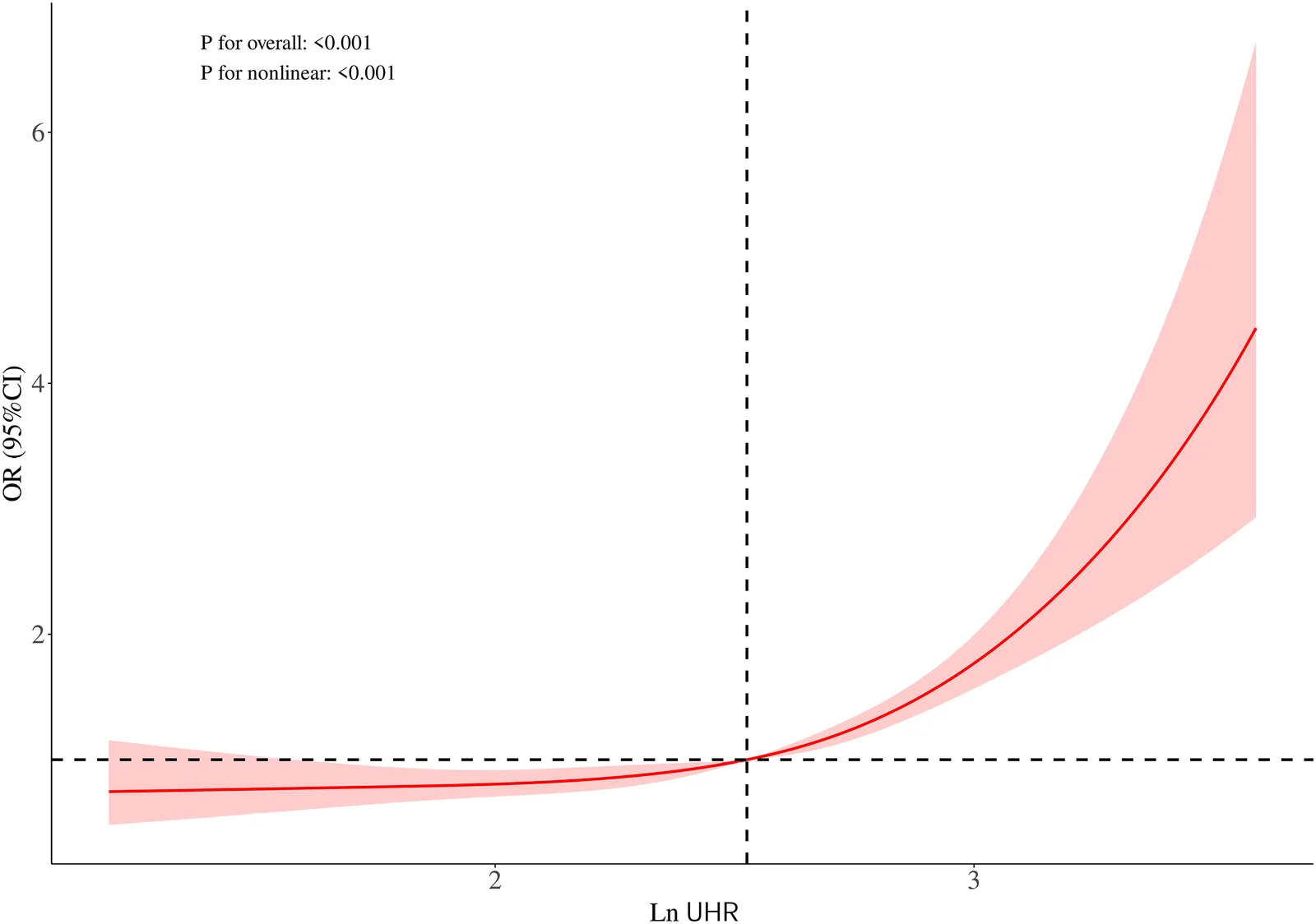
Survey-weighted restricted cubic spline of the dose–response relationship between ln-UHR and diabetic kidney disease (DKD). Model adjusted for gender, race, education, smoking, age, PIR, BMI, HbA1c, alcohol use, hypertension, albumin, ALT, BUN, and globulin. Shaded area represents 95% CI; *P*_overall < .001, *P* non-linear < .001. ALT = alanine transaminase, BMI = body mass index, BUN = blood urea nitrogen, CI = confidence interval, HbA1c= glycated hemoglobin, ln-UHR = the uric acid to high-density lipoprotein cholesterol ratio was logarithmically transformed using the natural logarithm (ln), OR = odds ratio, PIR = poverty income ratio, UHR = uric acid to high-density lipoprotein cholesterol ratio index.

### 3.3. Subgroup analyses

Table 3 presents the supplementary laboratory-adjusted subgroup analyses using the adjustment set specified in its legend. Point estimates were positive across the displayed subgroups, but their magnitudes and confidence intervals varied. No interaction test met the 2-sided 0.05 threshold (interaction *P* values .081–.894). These findings do not establish identical associations across subgroups.

### 3.4. Discriminative performance and model comparison

In the supplementary laboratory-inclusive model comparison, the base model had a survey-weighted area under the curve (AUC) of 0.75 (95% CI 0.73–0.77), compared with 0.76 (95% CI 0.74–0.78) after adding ln-UHR (Figs. 3 and 4). Bootstrap internal-validation AUCs were 0.72 (95% CI 0.69–0.75) and 0.73 (95% CI 0.70–0.76), respectively. The increase in apparent AUC was small; a paired test or CI for the AUC difference was not reported.

**Figure 3.**
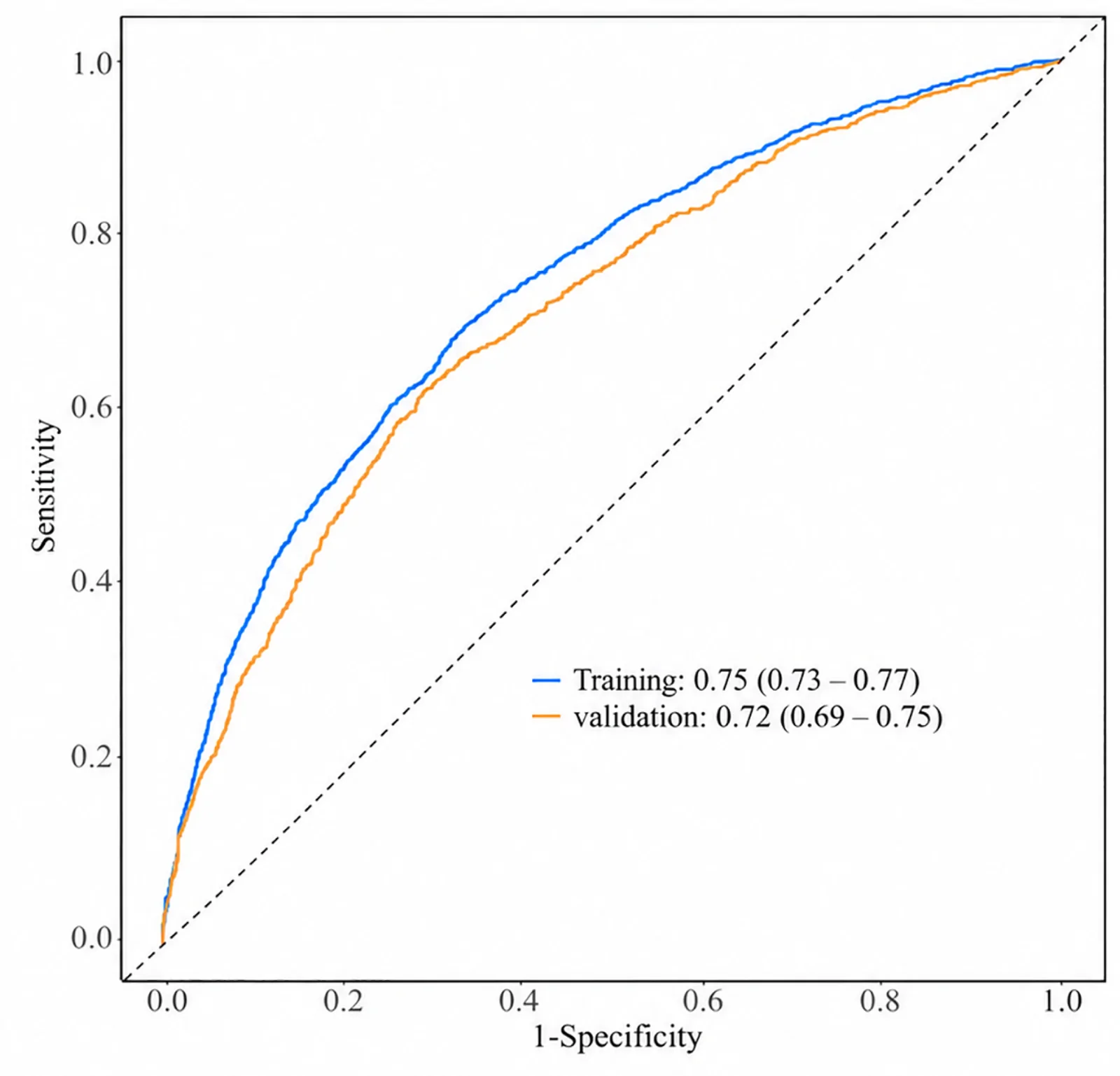
ROC curve of the baseline classification model. The base model contains the model 4 covariates without ln-UHR and is used for a separate exploratory discrimination comparison, rather than the primary association analysis. Survey-weighted AUC: 0.75 (95% CI 0.73–0.77); bootstrap internal-validation AUC: 0.72 (95% CI 0.69–0.75). Bootstrap denotes internal assessment, not an independent external validation cohort. AUC = area under the curve, CI = confidence interval, ln-UHR = the uric acid to high-density lipoprotein cholesterol ratio was logarithmically transformed using the natural logarithm (ln), ROC = receiver operating characteristic.

**Figure 4.**
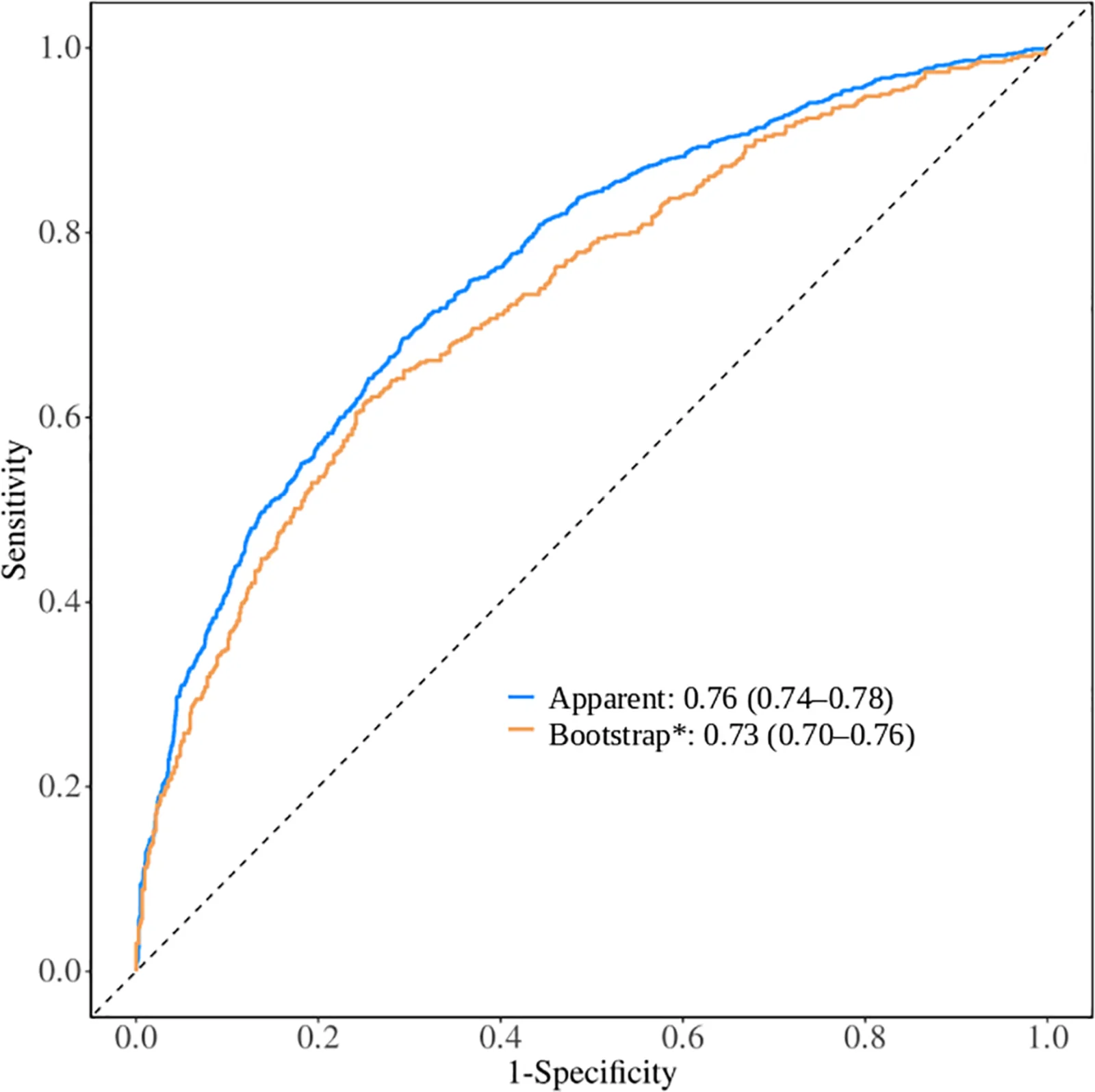
ROC curves after adding ln-UHR to the supplementary laboratory-inclusive base model. The extended model is the base model in Figure 3 plus ln-UHR. Survey-weighted AUC: 0.76 (95% CI 0.74–0.78); bootstrap internal-validation AUC: 0.73 (95% CI 0.70–0.76). Net reclassification improvement (NRI): 1.9% (95% CI 0.8%–3.1%). NRI was unweighted; its CI used unweighted bootstrap resampling (500 replicates, percentile method), with probability categories <10%, 10%–20%, and >20%. NRI is an exploratory sample-level measure, distinct from the survey-adjusted ROC analysis. AUC = area under the curve, CI = confidence interval, ln-UHR = the uric acid to high-density lipoprotein cholesterol ratio was logarithmically transformed using the natural logarithm (ln), ROC = receiver operating characteristic.

The exploratory unweighted category-based NRI was 1.9% (95% CI 0.8%–3.1%), using unweighted bootstrap resampling (500 replicates, percentile method). The calibration plot showed visual agreement between estimated and observed probabilities over much of the displayed range, with deviations near the upper end (Fig. 5). This graphical assessment was the principal calibration presentation. The Hosmer–Lemeshow test (*P* = .066) was reported as a secondary check and was not interpreted as proof of adequate calibration.

**Figure 5.**
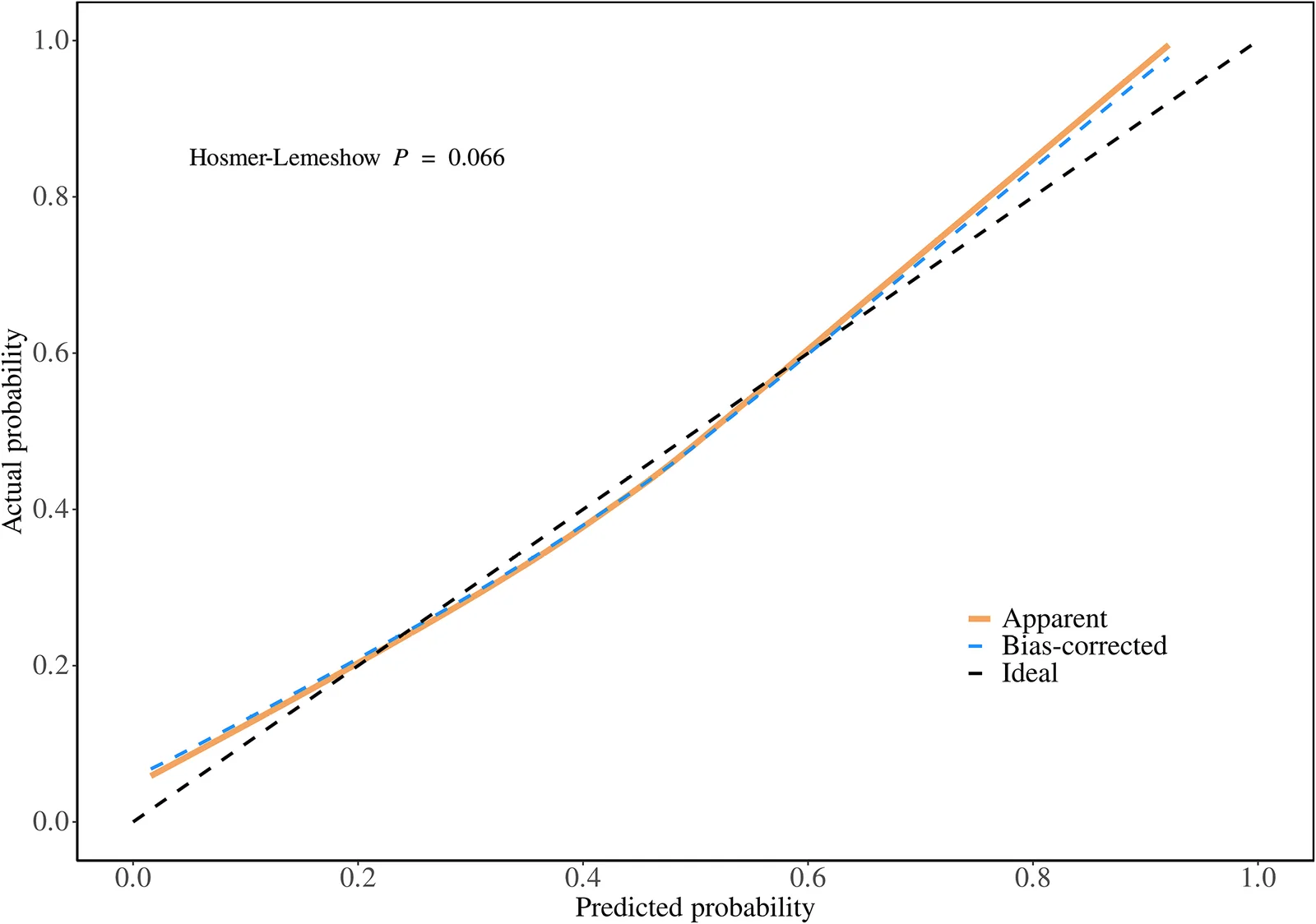
Calibration plot of the extended model (baseline + ln-UHR). *X*-axis = predicted probability, *Y*-axis = observed probability; solid line = apparent, dashed line = bias-corrected (500 bootstrap), diagonal = ideal. Hosmer–Lemeshow *P* = .066, reported as a secondary goodness-of-fit check only. ln-UHR = the uric acid to high-density lipoprotein cholesterol ratio was logarithmically transformed using the natural logarithm (ln).

## 4. Discussion

In this cross-sectional NHANES 2009 to 2018 analysis, higher UHR was associated with prevalent DKD in the primary confounder-adjusted model 3. *Q*4-versus-*Q*1 ORs were 2.43 for original DKD, 4.55 for strict DKD, and 2.82 for severe DKD. Model 4 yielded smaller estimates of 1.84, 2.78, and 1.93, respectively, and the severe-DKD comparison did not meet the 2-sided 0.05 threshold. The laboratory-adjusted spline supported a nonlinear association. More restrictive single-occasion DKD definitions do not establish persistent kidney injury.

Model 3 was emphasized because it adjusts for the selected potential confounders without the additional laboratory indicators whose temporal and causal roles are uncertain. Model 4 evaluates sensitivity to their inclusion. The observed attenuation is compatible with the hypothesized downstream roles of ALB, ALT, BUN, and GLB, but it is not specific evidence of mediation. Confounding, adjustment for consequences of kidney dysfunction, and the noncollapsibility of odds ratios also affect comparisons between differently adjusted logistic models. Accordingly, we did not calculate or interpret a proportion mediated from the change in ORs.

The separate laboratory-inclusive discrimination analysis showed a small increase in apparent AUC from 0.75 to 0.76, with internal-validation AUCs of 0.72 and 0.73. The unweighted NRI of 1.9% (95% CI 0.8%–3.1%) describes reclassification within the analyzed sample; it is not a survey-design-adjusted population estimate or evidence of clinical benefit. Calibration was interpreted primarily from the plotted agreement between estimated and observed probabilities, with the Hosmer–Lemeshow test serving as a supplementary check. These analyses concern classification of existing DKD rather than prediction of incident disease.

The global surge in obesity and diabetes epidemics has propelled DKD into a primary contributor to CKD progression and end-stage renal disease development across populations worldwide.^[29]^ Emerging as an independent predictor of diabetic microvascular sequelae, longitudinal cohort studies have demonstrated that obesity is a controllable factor in the etiology of multiorgan problems linked to diabetes, particularly through adipokine dysregulation and chronic inflammatory pathways.^[30]^ Several studies have shown that a higher BMI is positively correlated with a higher prevalence of DKD.^[31–33]^ Employing a longitudinal research design, investigators conducted time-dependent covariates analysis using Cox proportional hazards modeling to quantify dose–response relationships between longitudinally assessed cumulative UHR trajectories and both de novo CKD development and disease trajectory advancement, with rigorous adjustment for metabolic confounders.^[34]^ According to a study analyzing DKD risk prediction models across 20 cohorts, the chance of getting DKD increased by 16% for every 5 kg/m^2^ increase in body mass index.^[30]^ Gulali Aktas et al examined the possible link between high UHR and diabetic kidney injury (DKI). They examined data from 287 patients who had previously attended the outpatient clinic at their hospital as part of their retrospective analysis. The UHR values of diabetic individuals with and without DKI were studied. The study found that UHR had a strong connection with other renal function indicators and an independent predictive value for DKI, suggesting that it might be used as a diagnostic tool to detect renal damage.^[22]^ Utilizing data from NHANES, this population-based investigation analyzed 8817 participants to investigate the connection between insulin resistance and UHR levels. Elevated UHR was shown to have a substantial positive connection with insulin resistance indicators, indicating that it may be useful as a new biomarker for screening for insulin resistance in the US population.^[17,35]^ A different study showed that among diabetes individuals, increasing UHR is substantially and nonlinearly linked to higher all-cause and CVD mortality. Additionally, a U-shaped association between UHR and death from CVD and all causes was noted.^[35,36]^ In a recent retrospective analysis involving 343 hospitalized individuals with T2DM whose BMI is <24 kg/m^2^, the UHR was shown to have a strong correlation with NAFLD among patients who are neither overweight nor obese. This research suggests that UHR could be a useful and novel biomarker for NAFLD prediction.^[36,37]^

The observed UHR–DKD association can be discussed in the context of previously proposed relationships between UA, HDL, and kidney dysfunction. The present analysis did not test those mechanisms. Uric acid is mainly used as an antioxidant outside the cell, while it is a pro-oxidant inside the cell. In DKD, UA can contribute to endothelial cell dysfunction by promoting oxidative stress, thereby affecting renal function. The NOD-like receptor family pyrin domain containing 3 (NLRP3) inflammasome, a cytosolic multiprotein complex implicated in the etiology of certain inflammatory diseases, is activated as part of the pathophysiological cascade that UA starts, a cytosolic multiprotein complex implicated in the etiology of certain inflammatory diseases.starts. This molecular complex orchestrates the proteolytic processing and subsequent release of interleukin-1β, thereby amplifying inflammatory signaling cascades that contribute to the progression of DKD. Mechanistically, UA-induced NLRP3 inflammasome activation mediates renal fibrogenesis through sustained inflammatory microenvironment establishment, representing a critical pathway in DKD advancement. Furthermore, UA exerts direct pro-inflammatory effects on renal tubular epithelial cells, inducing phenotypic alterations that exacerbate tubular injury and perpetuate the inflammatory milieu, ultimately accelerating nephropathy progression. For example, utilizing allopurinol to lower UA levels can significantly reduce albuminuria and improve tubulointerstitial damage. UA may directly induce intercellular adhesion molecule-1 in cultured human renal proximal tubular epithelial cells. This molecule is an important mediator of inflammation.^[37]^ Emerging epidemiological research supports a pathophysiological continuity between increased blood UA concentrations and the pathophysiology of diabetes mellitus and its microvascular consequences, especially DKD. Mechanistic studies have elucidated that UA exacerbates renal deterioration through dual pathological pathways: overproduction of reactive oxygen species, which causes cellular macromolecules to oxidatively deteriorate and pro-inflammatory activation signaling cascades, including nuclear factor kappa B and NLRP3 inflammasome pathways, which collectively contribute to progressive glomerular and tubular injury in DKD.^[38]^ Emerging evidence implicates hyperuricemia as a significant contributor to CKD pathogenesis in T2DM populations. Furthermore, serum UA elevation demonstrates dose-dependent associations with progressive renal impairment, establishing it as an independent metabolic determinant of nephropathy advancement in diabetic cohorts.^[39]^ One new lipid biomarker for assessing atherosclerosis is the ratio of non-high-density lipoprotein cholesterol to high-density lipoprotein cholesterol (NHHR). NHHR may show the balance between these 2 kinds of lipoproteins by providing comprehensive information on beneficial and harmful lipoprotein particles. Previous research has shown that higher urine ALB excretion and an increased risk of chronic renal disease are linked to elevated NHHR.^[40]^ In patients with DKD, HDL exhibits higher levels of glycosylation, and its ability to promote cell migration is reduced compared with diabetic patients without kidney disease. The elevated risk of CVD in DKD patients may be partially explained by elevated glycated HDL levels, as these particles have a significantly reduced ability to stimulate endothelial cell migration.^[41]^ A meta-analysis and comprehensive review involving 7698 participants found that people with type 1 diabetes who had greater HDL-C levels had a decreased probability of developing DKD.^[42]^

This study has several strengths. First, we pooled 5 NHANES cycles (2009–2018) with full incorporation of the complex survey design (primary sampling units, strata, and cycle-adjusted MEC weights), so the estimates are representative of the noninstitutionalized U.S. adult population with T2DM. Second, covariate selection was guided by a prespecified directed acyclic graph, which allowed us to separate the primary confounder adjustment set (model 3) from laboratory indicators with uncertain causal roles (model 4), making the sensitivity of the association to adjustment transparent. Third, to our knowledge, this is the 1st study to examine the UHR–DKD association in a large, multi-ethnic U.S. sample, extending previous single-center and non-U.S. evidence. In addition, the prespecified strict and severe DKD definitions, the restricted cubic spline analysis, and the survey-weighted discrimination and calibration assessments were conducted to probe the robustness of the findings. These strengths notwithstanding, the study has several important limitations.

Several limitations warrant emphasis. The cross-sectional design does not establish whether higher UHR precedes DKD and does not exclude reverse causation. Single-occasion UACR and eGFR measurements cannot verify persistence for at least 3 months. Requiring both abnormalities or a higher UACR threshold does not resolve this temporal limitation. Although the model 3 contrasts were positive for all 3 definitions, the severe-DKD association did not meet the 2-sided 0.05 threshold after the additional model 4 adjustment.eGFR was estimated using the 2009 CKD-EPI equation without its race coefficient rather than the 2021 race-free equation; any resulting misclassification of eGFR <60 mL/min/1.73 m^2^ is independent of the UHR exposure and would be expected to bias estimates toward the null.

Residual confounding from diabetes duration, detailed medication use, and genetic predisposition remains unresolved. The DAG does not establish temporal ordering, and the primary adjustment set is not assumed to eliminate all confounding. Some model 4 measurements can be consequences or correlates of DKD; attenuation after their inclusion is not a formal mediation estimate. The unweighted NRI and its bootstrap interval do not incorporate NHANES stratification or clustering and are therefore limited to an exploratory sample-level interpretation.

The discrimination results concern prevalent DKD in the analyzed sample. Independent external validation is required to assess transportability, and prospective studies are needed to establish temporal relationships and assess future risk prediction. No clinical screening strategy or cutoff was validated in this study.

## 5. Conclusion

Higher UHR was associated with prevalent DKD among U.S. adults with T2DM in the primary model 3 analysis. Estimates were attenuated in the extended laboratory-adjusted model 4 sensitivity analysis. Supplementary analyses showed a small increase in apparent discrimination after adding ln-UHR. Prospective studies and independent external validation are needed before interpreting these findings as evidence for future risk prediction or clinical screening.

## Acknowledgments

The authors extend their sincere gratitude to all individuals who made valuable contributions to the preparation of this manuscript.

## Author contributions

**Data curation:** Wei Tan.

**Writing – original draft:** Ruolan Tan.

**Writing – review & editing:** Yulin Hu.



## References

[1] Guariguata L Whiting DR Hambleton I Beagley J Linnenkamp U Shaw JE. Global estimates of diabetes prevalence for 2013 and projections for 2035. Diabetes Res Clin Pract. 2014;103:137–49. 10.1016/j.diabres.2013.11.002 24630390

[2] Klein R Sharrett AR Klein BE. The association of atherosclerosis, vascular risk factors, and retinopathy in adults with diabetes: the atherosclerosis risk in communities study. Ophthalmology. 2002;109:1225–34. 10.1016/s0161-6420(02)01074-6 12093643

[3] Yau JW Rogers SL Kawasaki R. Global prevalence and major risk factors of diabetic retinopathy. Diabetes Care. 2012;35:556–64. 10.2337/dc11-1909 PMC3322721 22301125

[4] Anders HJ Huber TB Isermann B Schiffer M. CKD in diabetes: diabetic kidney disease versus nondiabetic kidney disease. Nat Rev Nephrol. 2018;14:361–77. 10.1038/s41581-018-0001-y 29654297

[5] Alicic RZ Rooney MT Tuttle KR. Diabetic kidney disease: challenges, progress, and possibilities. Clin J Am Soc Nephrol. 2017;12:2032–45. 10.2215/CJN.11491116 PMC5718284 28522654

[6] Selby NM Taal MW. An updated overview of diabetic nephropathy: diagnosis, prognosis, treatment goals and latest guidelines. Diabetes Obes Metab. 2020;22:3–15. 10.1111/dom.14007 32267079

[7] Mauer M Doria A. Uric acid and risk of diabetic kidney disease. J Nephrol. 2020;33:995–9. 10.1007/s40620-020-00796-z 32651849

[8] Macisaac RJ Ekinci EI Jerums G. Markers of and risk factors for the development and progression of diabetic kidney disease. Am J Kidney Dis. 2014;63:S39–62. 10.1053/j.ajkd.2013.10.048 24461729

[9] Johnson RJ Bakris GL Borghi C. Hyperuricemia, acute and chronic kidney disease, hypertension, and cardiovascular disease: report of a scientific workshop organized by the National Kidney Foundation. Am J Kidney Dis. 2018;71:851–65. 10.1053/j.ajkd.2017.12.009 PMC7286363 29496260

[10] Vareldzis R Perez A Reisin E. Hyperuricemia: an intriguing connection to metabolic syndrome, diabetes, kidney disease, and hypertension. Curr Hypertens Rep. 2024;26:237–45. 10.1007/s11906-024-01295-3 38270791

[11] Liu L Gao B Wang J. Time-averaged serum uric acid and 10-year incident diabetic kidney disease: a prospective study from China. J Diabetes. 2020;12:169–78. 10.1111/1753-0407.12983 31461212

[12] Ceriello A De Cosmo S Rossi MC. Variability in HbA1c, blood pressure, lipid parameters and serum uric acid, and risk of development of chronic kidney disease in type 2 diabetes. Diabetes Obes Metab. 2017;19:1570–8. 10.1111/dom.12976 28432733

[13] von Eckardstein A Nordestgaard BG Remaley AT Catapano AL. High-density lipoprotein revisited: biological functions and clinical relevance. Eur Heart J. 2023;44:1394–407. 10.1093/eurheartj/ehac605 PMC10119031 36337032

[14] Linton MF Yancey PG Tao H Davies SS. HDL function and atherosclerosis: reactive dicarbonyls as promising targets of therapy. Circ Res. 2023;132:1521–45. 10.1161/CIRCRESAHA.123.321563 PMC10213997 37228232

[15] Russo GT De Cosmo S Viazzi F. Plasma triglycerides and HDL-C levels predict the development of diabetic kidney disease in subjects with type 2 diabetes: the AMD annals initiative. Diabetes Care. 2016;39:2278–87. 10.2337/dc16-1246 27703024

[16] Wang H Wu J Lin M Hu Y Ma Y. High levels of high-density lipoprotein cholesterol may increase the risk of diabetic kidney disease in patients with type 2 diabetes. Sci Rep. 2024;14:15362. 10.1038/s41598-024-66548-2 PMC11224420 38965304

[17] Xie Y Huang K Zhang X. Association of serum uric acid-to-high-density lipoprotein cholesterol ratio with non-alcoholic fatty liver disease in American adults: a population-based analysis. Front Med (Lausanne). 2023;10:1164096. 10.3389/fmed.2023.1164096 PMC10225665 37256087

[18] Li Z Liu Q Yao Z. The serum uric acid-to-high-density lipoprotein cholesterol ratio is a predictor for all-cause and cardiovascular disease mortality: a cross-sectional study. Front Endocrinol (Lausanne). 2024;15:1417485. 10.3389/fendo.2024.1417485 PMC11427315 39345882

[19] Yang Y Zhang J Jia L Su J Ma M Lin X. Uric acid to high-density lipoprotein cholesterol ratio predicts adverse cardiovascular events in patients with coronary chronic total occlusion. Nutr Metab Cardiovasc Dis. 2023;33:2471–8. 10.1016/j.numecd.2023.07.037 37586923

[20] Aktas G Yilmaz S Kantarci DB. Is serum uric acid-to-HDL cholesterol ratio elevation associated with diabetic kidney injury? Postgrad Med. 2023;135:519–23. 10.1080/00325481.2023.2214058 37170820

[21] Park B Jung DH Lee YJ. Predictive value of serum uric acid to HDL cholesterol ratio for incident ischemic heart disease in non-diabetic Koreans. Biomedicines. 2022;10:1672. 10.3390/biomedicines10061422 PMC9219787 35740443

[22] Zhou X Xu J. Association between serum uric acid-to-high-density lipoprotein cholesterol ratio and insulin resistance in an American population: a population-based analysis. J Diabetes Investig. 2024;15:762–71. 10.1111/jdi.14170 PMC11143423 38407574

[23] Zipf G Chiappa M Porter KS Ostchega Y Lewis BG Dostal J. National health and nutrition examination survey: plan and operations, 1999–2010. Vital Health Stat 1. 2013;56:1–37. 25078429

[24] Stevens PE Levin A. Evaluation and management of chronic kidney disease: synopsis of the kidney disease: improving global outcomes 2012 clinical practice guideline. Ann Intern Med. 2013;158:825–30. 10.7326/0003-4819-158-11-201306040-00007 23732715

[25] Chen TK Knicely DH Grams ME. Chronic kidney disease diagnosis and management: a review. JAMA. 2019;322:1294–304. 10.1001/jama.2019.14745 PMC7015670 31573641

[26] Levey AS Stevens LA Schmid CH. A new equation to estimate glomerular filtration rate. Ann Intern Med. 2009;150:604–12. 10.7326/0003-4819-150-9-200905050-00006 PMC2763564 19414839

[27] Rognant N Lemoine S Laville M Hadj-Aïssa A Dubourg L. Performance of the chronic kidney disease epidemiology collaboration equation to estimate glomerular filtration rate in diabetic patients. Diabetes Care. 2011;34:1320–2. 10.2337/dc11-0203 PMC3114318 21540431

[28] Pichler R Afkarian M Dieter BP Tuttle KR. Immunity and inflammation in diabetic kidney disease: translating mechanisms to biomarkers and treatment targets. Am J Physiol Renal Physiol. 2017;312:F716–31. 10.1152/ajprenal.00314.2016 PMC6109808 27558558

[29] Hu J Yang S Zhang A. Abdominal obesity is more closely associated with diabetic kidney disease than general obesity. Diabetes Care. 2016;39:e179–80. 10.2337/dc16-1025 27538755

[30] Gray N Picone G Sloan F Yashkin A. Relation between BMI and diabetes mellitus and its complications among US older adults. South Med J. 2015;108:29–36. 10.14423/SMJ.0000000000000214 PMC4457375 25580754

[31] Tanaka S Tanaka S Iimuro S. Maximum BMI and microvascular complications in a cohort of Japanese patients with type 2 diabetes: the Japan Diabetes Complications Study. J Diabetes Complications. 2016;30:790–7. 10.1016/j.jdiacomp.2016.02.020 26997170

[32] Polemiti E Baudry J Kuxhaus O. BMI and BMI change following incident type 2 diabetes and risk of microvascular and macrovascular complications: the EPIC-Potsdam study. Diabetologia. 2021;64:814–25. 10.1007/s00125-020-05362-7 PMC7940263 33452586

[33] Jiang W Wang J Shen X. Establishment and validation of a risk prediction model for early diabetic kidney disease based on a systematic review and meta-analysis of 20 cohorts. Diabetes Care. 2020;43:925–33. 10.2337/dc19-1897 32198286

[34] Liu P Li J Yang L. Association between cumulative uric acid to high-density lipoprotein cholesterol ratio and the incidence and progression of chronic kidney disease. Front Endocrinol (Lausanne). 2023;14:1269580. 10.3389/fendo.2023.1269580 PMC10753577 38155948

[35] Ding L Guo H Zhang C. Serum uric acid to high-density lipoprotein cholesterol ratio is a predictor for all-cause and cardiovascular disease mortality in patients with diabetes: evidence from NHANES 2005–2018. Nutr Metab Cardiovasc Dis. 2024;34:2480–8. 10.1016/j.numecd.2024.07.001 39174432

[36] Cui Y Qu Z Hu W Shi H. Relationship between uric acid to high density lipoprotein cholesterol ratio and nonalcoholic fatty liver disease in nonoverweight/obese patients with type 2 diabetes. Int J Endocrinol. 2023;2023:2513175. 10.1155/2023/2513175 PMC10409575 37560201

[37] Wang M Lin X Yang X Yang Y. Research progress on related mechanisms of uric acid activating NLRP3 inflammasome in chronic kidney disease. Ren Fail. 2022;44:615–24. 10.1080/0886022X.2022.2036620 PMC9004527 35382689

[38] Bjornstad P Lanaspa MA Ishimoto T. Fructose and uric acid in diabetic nephropathy. Diabetologia. 2015;58:1993–2002. 10.1007/s00125-015-3650-4 PMC4826347 26049401

[39] Yan D Tu Y Jiang F. Uric acid is independently associated with diabetic kidney disease: a cross-sectional study in a Chinese population. PLoS One. 2015;10:e0129797. 10.1371/journal.pone.0129797 PMC4451151 26029914

[40] Jiang J Zhou X Lan L Weng J Ren W. The correlation between serum uric acid and diabetic kidney disease in adult-onset type 1 diabetes patients in China. Acta Diabetol. 2023;60:1231–9. 10.1007/s00592-023-02119-7 PMC10359385 37264251

[41] Pan J Li C Zhang J. Association between non-high-density lipoprotein cholesterol to high-density lipoprotein cholesterol ratio (NHHR) and diabetic kidney disease in patients with diabetes in the United States: a cross-sectional study. Lipids Health Dis. 2024;23:317. 10.1186/s12944-024-02308-5 PMC11437633 39334123

[42] Rysz J Gluba-Brzózka A Rysz-Górzyńska M Franczyk B. The role and function of HDL in patients with chronic kidney disease and the risk of cardiovascular disease. Int J Mol Sci. 2020;21:601. 10.3390/ijms21020601 PMC7014265 31963445

